# A computational approach to identify efficient RNA cleaving 10–23 DNAzymes

**DOI:** 10.1093/nargab/lqac098

**Published:** 2023-01-10

**Authors:** Angela C Pine, Greg N Brooke, Antonio Marco

**Affiliations:** School of Life Sciences, University of Essex, Wivenhoe Park, Colchester CO4 3SQ, UK; School of Life Sciences, University of Essex, Wivenhoe Park, Colchester CO4 3SQ, UK; School of Life Sciences, University of Essex, Wivenhoe Park, Colchester CO4 3SQ, UK

## Abstract

DNAzymes are short pieces of DNA with catalytic activity, capable of cleaving RNA. DNAzymes have multiple applications as biosensors and in therapeutics. The high specificity and low toxicity of these molecules make them particularly suitable as therapeutics, and clinical trials have shown that they are effective in patients. However, the development of DNAzymes has been limited due to the lack of specific tools to identify efficient molecules, and users often resort to time-consuming/costly large-scale screens. Here, we propose a computational methodology to identify 10–23 DNAzymes that can be used to triage thousands of potential molecules, specific to a target RNA, to identify those that are predicted to be efficient. The method is based on a logistic regression and can be trained to incorporate additional DNAzyme efficiency data, improving its performance with time. We first trained the method with published data, and then we validated, and further refined it, by testing additional newly synthesized DNAzymes in the laboratory. We found that although binding free energy between the DNAzyme and its RNA target is the primary determinant of efficiency, other factors such as internal structure of the DNAzyme also have an important effect. A program implementing the proposed method is publicly available.

## INTRODUCTION

DNAzymes or deoxyribozymes are catalytically active single-stranded DNA molecules (1). Multiple types of DNAzyme exist, with different applications, such as DNA and RNA ligation/cleavage/splicing, DNA phosphorylation, peroxidase and photolyase activity (2–5). In addition to oligonucleotides, DNAzymes can also react with other substrates such as porphyrin and hemin (6–8). DNAzymes have been developed as therapeutics, diagnostic tools and biosensors for metal ions and bacteria (3,9–11). They have even been used for logic gates (12,13), computing circuits and switches (14) and in the catalysis of biofuel cells (15). This oligonucleotide technology is therefore being developed for a broad range of applications.

Here, we focus on the ‘10–23’ DNAzyme, an RNA-cleaving molecule, consisting of a 15-nucleotide catalytic core flanked by substrate binding arms of variable length and sequence that confer specificity by interacting with target RNA via Watson–Crick base pairing interactions (16). These DNAzymes cleave RNA between an unpaired purine (A, G) and a paired pyrimidine (U, C) in the presence of a divalent cation, such as Mg^2+^, via a de-esterification reaction (17,18). This results in phosphodiester bond cleavage of the target and generation of two RNA fragments (16). The recently published high-resolution nuclear magnetic resonance characterization of a 10–23 DNAzyme revealed that these reactions consist of multiple rate-limiting transient intermediate states and that the DNAzyme has conformational plasticity (19). Due to their specificity and low immunogenicity, these DNAzymes have been progressed through to clinical trials and are being developed for multiple diseases such as asthma (20) and cancer (21). For example, a 10–23 DNAzyme targeting GATA-3 (SB010) has undergone clinical trials for the treatment of chronic obstructive pulmonary disease. SB010 was found to be well tolerated and to successfully reduce airway inflammation (22), demonstrating the potential of DNAzymes as therapeutics.

DNAzymes belong to a family of antisense molecules, including ribozymes, small interfering RNA (siRNA) and short hairpin RNA (shRNA). DNAzymes have several advantages over RNA-based reagents, being more cost-effective and stable (23). However, RNA-based technologies have become more established than DNAzymes and this is, at least in part, as a result of the differences in the availability of online user-friendly scan tools. In the case of siRNA, for example, a number of critical criteria have been identified and multiple tools exist to aid in the identification of these molecules (24). These tools are often capable of identifying binding sites with suitable characteristics (e.g. binding energy and specific sequence conditions) meaning sequences predicted to have off-target effects can be eliminated (25).

The use of computational tools for the identification of 10–23 DNAzymes is currently limited. As a result, researchers often have to perform large costly and time-consuming screens to identify lead molecules. The lack of tools has hampered the development of this technology, and hence there is a great need for computational methods to aid in DNAzyme design. Currently available bioinformatics sites and software are mainly limited to ‘oligo analysers’, such as those provided by oligo suppliers that are primarily used for primer design. These tools can be used to aid in the prediction of inter/intramolecular structures in DNAzymes but are limited in their suitability for the assessment of other parameters. A recent advancement in the field has been the development of DNAzyme repositories (26) and DNAzyme selectors (27), providing data from published cleavage reactions. However, tools for the identification and efficiency prediction of custom DNAzymes are still missing.

Here, we employed the use of other online packages to facilitate the identification of DNAzymes (such as those provided by ViennaRNA Web Services or the Frieburg RNA Tools), despite them mainly catering for duplex formation between RNA molecules rather than the heteroduplex (RNA:DNA) seen with these functional oligonucleotides (28,29). The lack of relevant computational tools has resulted in the identification of DNAzymes being largely a manual, stepwise process consisting of the identification of accessible sites for antisense oligonucleotides by techniques such as ‘messenger walk screening’ or RNase H assays to identify accessible sites for antisense oligonucleotides (30). This is then followed by *in vitro* cleavage reactions and possibly the consideration of a small number of parameters that have been described to be important in DNAzyme efficiency.

**Figure 1.**
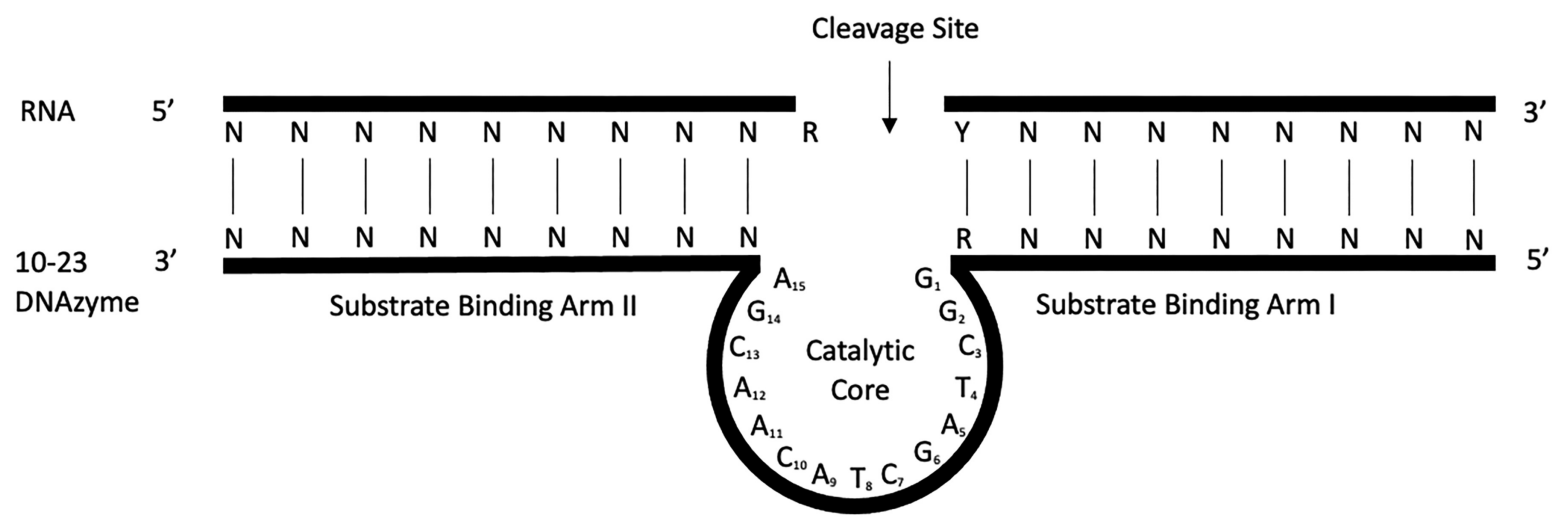
The structure of the 10–23 DNAzyme with 9 nt substrate binding arms. RNA transcript that acts as substrate is the top strand with an RY cleavage site (R represents A or G and Y is C or U). Arms I and II are the substrate binding arms that complement the RNA via Watson–Crick base pairing.

The purine:pyrimidine junction (Figure 1), necessary for 10–23 DNAzyme target cleavage, is usually the primary parameter considered when designing DNAzymes. Scanning of a target mRNA for these bases will result in tens to hundreds of potential cleavage sites, depending on the size and sequence of the transcript. Studies by Cairns *et al.* have provided further insight into the cleavage efficiency of different purine:pyrimidine junctions, with the efficiency of the different combinations being AU = GU GC >>AC, where AU/GU junctions prove most efficient followed by GC and lastly AC (28,29). The number of potential DNAzymes can then be reduced by selecting only those found in regions of interest in the transcript and by considering parameters such as the stability of the DNAzyme–RNA interaction or the formation of internal structures in the DNAzyme.

The stability of nucleic acid interactions or structural features is typically measured as the free energy (Gibbs) resulting from the pairing of nucleotides and is often referred to as Δ*G*. Δ*G* estimations for DNA/RNA interactions were first described by Sugimoto *et al.* using the nearest-neighbour model (31). This model suggests that the identity and orientation of nucleotides neighbouring each other form base pair interactions contributing to stability and that this should be taken into account rather than treating the DNAzyme or RNA as a string of interactions (32). The Δ*G* in this context specifically refers to the free energy required to unwind the heteroduplex (31). The formation of intramolecular structures or hairpins was suggested as another possible parameter important in determining DNAzyme efficiency in the study by Cairns. These hairpins form when two regions of the same DNAzyme are complementary in their sequence and bind to form a double helix with an unpaired loop at the end (31). The free energy of binding can be determined for hairpin formation and indicates the energy required to break the secondary structure. Similarly, duplexes between two DNAzyme molecules (dimer formation) can also affect DNAzyme efficiency. Due to the lack of tools to aid DNAzyme design, we have developed a computational approach that considers parameters that might affect DNAzyme activity to identify optimal binding arms to facilitate efficient target cleavage. We believe that this tool will significantly reduce the time and costs associated with the identification of DNAzymes, facilitating the rapid progression of these molecules as, for example, therapeutics and biosensors.

## MATERIALS AND METHODS

### Oligonucleotides

Oligonucleotides were synthesized by Sigma Aldrich (MI, USA). All DNAzymes were constructed using the ‘10–23’ catalytic core motif (5′-GGCTAGCTACAACGA-3′) and nine nucleotide substrate binding arms.

### *In vitro* transcription

The HPV16 E6/E7 RNA transcript was *in vitro* transcribed from a template plasmid containing an upstream T7 promoter sequence (synthesized by Twist Bioscience, San Francisco, USA). Prior to *in vitro* transcription, the plasmid was linearized using a 3′ NotI restriction site. *In vitro* transcription was performed on 1 μg of purified linearized plasmid DNA using the TranscriptAid High Yield T7 RNA polymerase kit (ThermoFisher Scientific, Paisley, UK) and fluorescein-12-dUTPs for labelling (Roche, St Albans, UK), following the manufacturer’s instructions. RNA was purified using a RNeasy MinElute Cleanup Kit (Qiagen, Hilden, Germany), and RNA length, quality and yield were checked using gel electrophoresis and a nanodrop spectrophotometer.

### DNAzyme cleavage reactions

Individual DNAzymes and RNA substrate, at a ratio of 10:1 (in μM), were incubated separately at 37°C for 10 min in equal volumes of reaction buffer (50 mM Tris-HCl, pH 7.5, 150 mM NaCl, 10 mM MgCl_2_, 0.01% sodium dodecylsulphate) (28). The DNAzyme and RNA substrate were mixed and incubated at 37°C for 60 min. Reactions were stopped by the addition of an equal volume of RNA loading dye (New England Biolabs, Ipswich, UK) and snap freezing.

### Urea polyacrylamide gel electrophoresis

Samples were boiled at 70°C for 10 min and cooled on ice for 2 min prior to electrophoresis on 5% denaturing urea polyacrylamide gels (run at 120–150 V for 1 h). Gels were imaged using the Fusion FX Imager (Vilber Lourmat, Collégien, France) and band intensity measured using ImageJ v1.52k (33). The relative intensity of product bands, as a proportion of the total intensity of all bands, was calculated.

### Prediction of thermodynamic properties of DNAzymes

Potential DNAzyme cleavage sites were identified in the HPV16 E6/E7 transcript sequence (accession number: MH937393) by looking for purine:pyrimidine (RY) junctions. The initial scan of HPV16 E6/E7 identified 205 potential target sites. DNAzymes containing either GC or AC purine:pyrimidine junctions were excluded from further screening, as it is already known that these are the least efficient DNAzymes (28). DNAzymes were then constructed using nine nucleotide substrate binding arms on either side of the catalytic core. Logistic regressions were built as generalized linear models with binomial probability distribution and logit link function. The logistic regression framework allows for the evaluation of the relative contribution of multiple factors as independent variables to explain the cleaving efficiency as a dependent variable.

Pairing energies were predicted, assuming perfect pairing between the target and the DNAzyme arms, with the nearest-neighbour method using the energies for RNA:DNA pairs measured by Sugimoto *et al.* (31). Energies of predicted internal structures were computed with RNAfold, corresponding to the predicted structure of the lowest energy. Pairing energies for potential homodimers were obtained with RNAcofold, as the pairing free energy of the longest possible paired segment. Both RNAfold and RNAcofold are from the Vienna RNA package (34), using the DNA pairing energies as provided by Mathews *et al* (35). All statistical analyses were done with R 3.6.3.

## RESULTS

### Relationship between binding energy and DNAzyme efficiency

**Figure 2.**
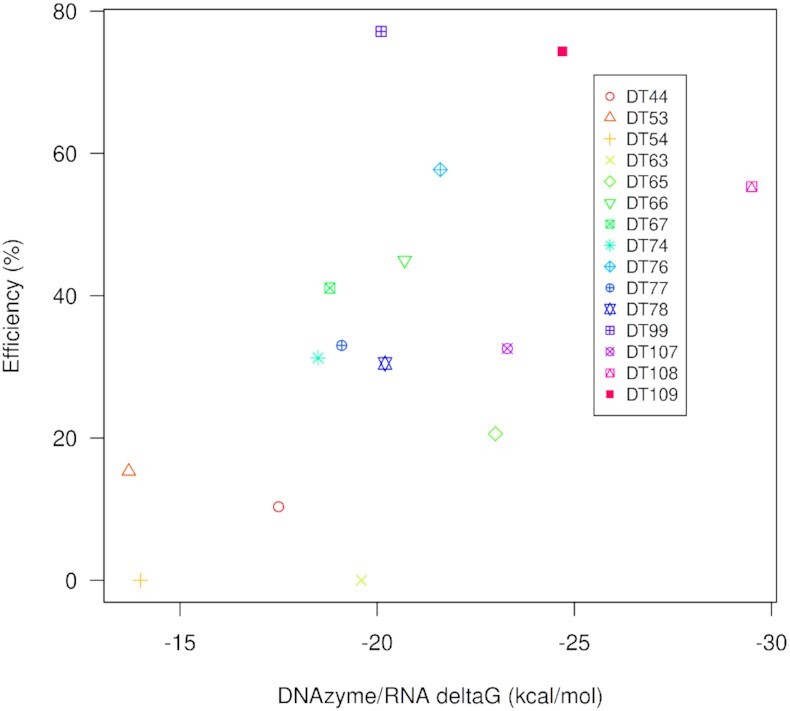
Efficiency and free energy of DNAzyme/RNA interactions. Scatter plot of the Δ*G* (free energy) of the interaction between published DNAzyme and their putative RNA targets and the efficiency measured in the lab and percent of degraded subtract after 60 min.

Several parameters have been proposed to affect DNAzyme efficiency, for example, internal structures and hybridization free energy/binding interaction. The strength of the binding interaction between a DNAzyme and its target is directly related to the efficiency of the cleavage reaction (28). In order to investigate this relationship, we experimentally assessed the cleavage efficiency of 15 previously published 10–23 DNAzymes targeting HPV16 E6/E7 (Supplementary Figures S1a and S2a) and compared this with their predicted binding energies from thermodynamic models (Table 1). Cleavage efficiency of the DNAzymes was quantified under single turnover conditions (a 10-fold excess of DNAzyme over substrate RNA) after a 60-min incubation at 37°C (Supplementary Figure S1). Our binding energy predictions differed slightly from those previously reported (28); however, the differences were minimal (Table 1; *r* = 0.994, *P* < 0.0001). We observed that DNAzyme efficiency has a sigmoid-like distribution, where high binding energy interactions (i.e. less stable) have low efficiencies and vice versa, where the transition between low and high efficiencies is approximately −20 kcal/mol (Figure 2). This suggests that logistic models are particularly suitable to predict DNAzyme efficiency based on predicted binding energy.

**Table 1.** Published DNAzymes cleaving HPV16 (E6/E7) RNA

Dz*	Sequence**	Energy (kcal/mol)	Internal (kcal/mol)	Dimer (kcal/mol)	Efficiency ± SEM (%)
DT44	GTTGTTCCA[CC]ACAAACTAT	−17.5	−2.7	−8.3	10.3 ± 10.3
DT53	CTATACTCA[CC]TAATTTTAG	−13.7	0	−7.7	15.3 ± 15.3
DT54	ACTCACTAA[CC]TTTAGAATA	−14	0	−7.4	0 ± 0
DT63	CATACAGCA[CC]ATGGATTCC	−19.6	−0.7	−6.9	0 ± 0
DT65	GCATATGGA[CC]TCCCATCTC	−23	−1.1	−6.7	20.6 ± 7.4
DT66	GGATTCCCA[CC]CTCTATATA	−20.7	−0.2	−7.8	45 ± 2.6
DT67	CCATCTCTA[CC]ATACTATGC	−18.8	0	−7.4	41.1 ± 4.2
DT74	AAAGTCATA[CC]ACCTCACGT	−18.5	−0.9	−6.8	31.2 ± 9.1
DT76	TATACCTCA[CC]GTCGCAGTA	−21.6	−0.7	−6.2	57.7 ± 2.3
DT77	TCGCAGTAA[CC]TGTTGCTTG	−19.1	−3.4	−4.6	33 ± 6.9
DT78	CTTGCAGTA[CC]ACACATTCT	−20.2	−1.9	−5.9	30.5 ± 15.2
DT99	GTTTCTCTA[CC]GTGTTCTTG	−20.1	−0.2	−7.7	77.1 ± 3.2
DT107	ATACATCGA[CC]CGGTCCACC	−23.3	−0.4	−7.8	32.6 ± 5.7
DT108	ACCGGTCCA[CC]CGACCCCTT	−29.5	−0.2	−7.8	55.3 ± 4.6
DT109	GTCCACCGA[CC]CCCTTATAT	−24.7	0	−7	74.3 ± 2.6

*DNAzyme; **[CC] : 10–23 DNAzyme catalytic core (GGCTAGCTACAACGA)

**Figure 3.**
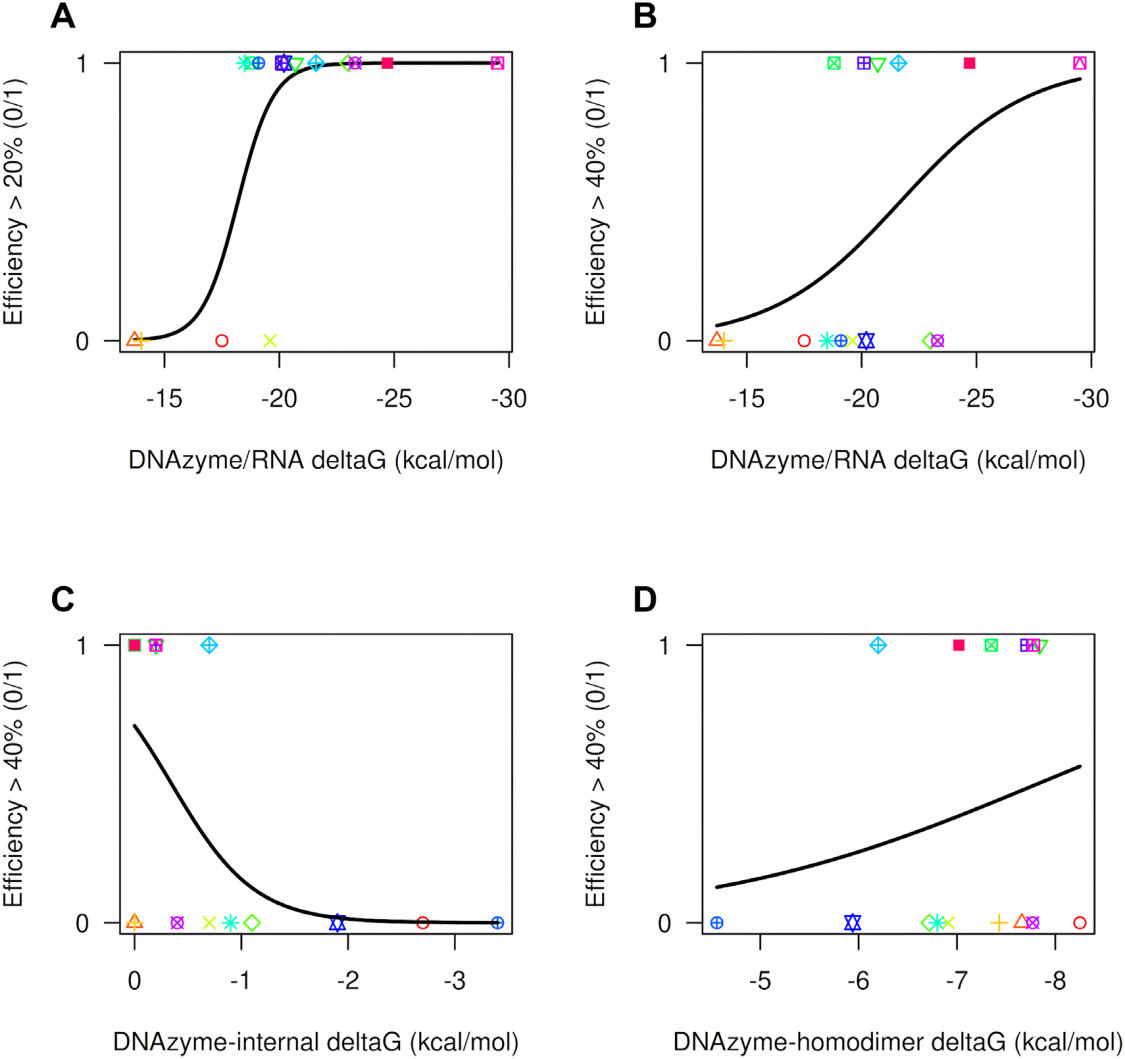
Logistic fitted lines for energy features versus successful cleavage. (**A**) Δ*G* (free energy) of the DNAzyme–RNA interaction against cleavage success using a threshold of 20% of efficiency. (**B**) As panel (A) but using a threshold of 40%. (**C**) Internal free energy of the DNAzyme molecule versus success. (**D**) Homodimer formation free energy versus success. Color code as in Figure 1 panel.

We first chose an efficiency threshold of 20% to define whether a DNAzyme is cleaving a target and fitted a logistic curve (Figure 3A, *P* = 0.0015). As expected, very high energy interactions were associated with cleavage in the model. However, some of these DNAzymes had poor efficiency. Hence, we fitted a second logistic curve considering a good efficiency when the DNAzyme cleaves over 40% of the target DNA within an hour (Figure 3B, *P* = 0.01342). In this case, although the fit was good, relatively low binding energies associated with some of the DNAzymes corresponded with low efficiencies (notably DT65, DT107 and HPV5). In agreement with others, binding energy alone is not sufficient to predict DNAzyme efficiency.

### Relationship between internal structures/homodimers and DNAzyme efficiency

Binding energy alone was not sufficient to predict DNAzyme efficiency. This led us to explore the role of other variables that may affect efficiency. First, we fitted a logistic regression for efficiencies >40% using only the free energy of internal forming structures (i.e. hairpins) with RNAcofold (34, see Materials and Methods), and we found a significant fit, where the lower (more stable) the energy of potential structures, the lower the predicted efficiency (Figure 3C, *P* = 0.01824). To see if homodimers affect DNAzyme efficiency, we also fitted a model using the hybridization energy of potential homodimers as the only variable. In contrast to hairpins, regression analysis of the free energy of predicted DNAzyme homodimers was not statistically significant (Figure 3D, *P* = 0.3592). It therefore appears that hairpin formation, but not homodimers, has an effect upon DNAzyme efficiency.

### A model to predict DNAzyme efficiency

To account for multiple parameters affecting DNAzyme efficiency, we built a general multiple logistic regression model incorporating, in addition to binding energy, two other parameters: dimer formation energy and internal structure energy. The former is a measure of potential homodimers forming during the cleavage reaction, and the latter considers internal structures (mostly hairpins) that the DNAzyme may have. In agreement with the previous results, binding energy and internal structure energy were associated with DNAzyme efficiency, and we did not find a significant association with dimer formation energy (Table 2).

**Table 2.** Logistic regression of thermodynamic properties and efficiencies for published DNAzymes

	df	Deviance	Residual df	Residual deviance	*P*-value
Null			14	20.2	
Binding energy	1	4.4	13	16.1	0.0436
Dimer energy	1	0.9	12	15.2	0.3352
Internal energy	1	15.2	11	0.0	<0.001

To assess our model, we synthesized 15 additional, randomly chosen novel DNAzymes targeting HPV16 E6/E7 and performed cleavage reactions on full-length transcripts (Table 3, named HPV5-29). These DNAzymes were selected as they were predicted to have a good range of efficiencies. For consistency, we used a substrate binding arm length of 9 nt, which we found to be efficient in cleavage reactions that compared different substrate binding arm lengths (Supplementary Figure S3 and Table S1). Cleavage efficiency of these new DNAzymes was quantified under single turnover conditions after a 60-min incubation at 37°C (Supplementary Figures S2b and S3). The DNAzymes were classified into high and low efficiency (40% threshold) and two prediction models were evaluated to see which would perform better at predicting DNAzyme efficiency: binding energy only (the 1E15 model, Table 4) and binding energy, internal structure and dimer formation energies (the 3E15 model, Table 4). We obtained the best combination of accuracy and precision from the single model (73.3% and 25% respectively, Table 4), but the multiple energies model has a similar performance despite the limited number of data points to fit three parameters. Further, it is possible that the addition of more data to the multiple energies model may result in this method outperforming the single energy model. The number of true and false positives and negatives are listed in Supplementary Table S2.

**Table 3.** DNAzymes newly synthesized in this study

Dz*	Sequence**	Energy (kcal/mol)	Internal (kcal/mol)	Dimer (kcal/mol)	Efficiency ± SEM (%)
HPV5	TTCAGGACA[CC]AGTGGCTTT	−20.7	−1.9	−5	6.9 ± 6.9
HPV7	AGACATACA[CC]CGACCGGTC	−21.7	−0.6	−8.8	0 ± 0
HPV12	CAAGACATA[CC]ATCGACCGG	−18.7	0	−7.3	29 ± 11.8
HPV15	TCTTCAGGA[CC]ACAGTGGCT	−21.8	−1.9	−4.7	45.5 ± 3.6
HPV17	TACAGCATA[CC]GGATTCCCA	−22	−1.5	−6.3	0 ± 0
HPV20	TATCACATA[CC]AGCATATGG	−16.3	−3.5	−3.8	0 ± 0
HPV21	TTTATCACA[CC]ACAGCATAT	−16.5	−0.6	−6.3	13.9 ± 13.9
HPV22	ATGTCTATA[CC]TCACTAATT	−15.4	0	−6.8	21.6 ± 10.9
HPV23	TAATGTCTA[CC]ACTCACTAA	−15	−0.1	−7.9	9.1 ± 9.1
HPV24	CTGTGGTAA[CC]TTTCTGGGT	−20.8	0	−7.1	62.2 ± 3.1
HPV25	TTGTCCAGA[CC]GTCTTTGCT	−22.6	−0.3	−6.5	28.8 ± 8.6
HPV26	TTTGTTGTA[CC]TGCTGTTCT	−19.9	−5.8	−5.7	0 ± 0
HPV27	TGTTCTTGA[CC]GATCTGCAA	−19.2	0	−7.1	0 ± 0
HPV28	CAGTAGAGA[CC]CAGTTGTCT	−19.7	−3.4	−7	22.8 ± 4.9
HPV29	ATATTGTAA[CC]GGGCTCTGT	−19.8	−1.2	−8.4	22.1 ± 0.1
HPV30	GAGAACAGA[CC]GGGGCACAC	−21.4	0	−6.6	0 ± 0.0
HPV31	CTGTTCTAA[CC]GTTGTTCCA	−20.4	−0.8	−7.1	44.8 ± 3
HPV33	GAGCTGTCA[CC]TTAATTGCT	−19.7	−1.6	−4.8	0 ± 0.0
HPV34	GACCATCTA[CC]TTCATCCTC	−21.6	−0.3	−7.1	41.8 ± 0.8
HPV35	TACGCACAA[CC]CGAAGCGTA	−20	−1.6	−7.4	0 ± 0.0
HPV36	GAATGTCTA[CC]GTGTGTGCT	−20.6	−1.6	−6.1	45.8 ± 3.9
HPV37	TGCTTTGTA[CC]GCACAACCG	−21.7	−6.2	−4.2	0 ± 0.0
HPV38	GCCCATTAA[CC]AGGTCTTCC	−23.3	−1.5	−5.2	22.2 ± 0.5

^a^DNAzyme; ^b^[CC] : 10–23 DNAzyme catalytic core (GGCTAGCTACAACGA)

**Table 4.** Performance of the logistic regression to predict DNAzyme efficiency

	1E15^a^	3E15^b^	3E30^c^
Accuracy	73.3%	66.7%	62.5%
Precision	25.0%	20.0%	50.0%
Recall	50.0%	50.0%	33.0%

^a^One energy model, 15 DNAzymes for training and 15 DNAzymes for validation; ^b^Three energies model, 15 for training and 15 for validation; ^c^Three energies model, 30 for training and 8 for validation. Efficiency thresholds were set to 40%.

To investigate whether more data will improve the logistic regressions used for predicting DNAzyme efficiencies, we included our novel DNAzymes into a new logistic regression, resulting in a total of 30 DNAzymes with measured efficiencies. This model had strong statistical support for a contribution of binding energy and internal structure energy in the prediction outcome (Table 5). To independently assess this model, we synthesized 8 more DNAzymes, in addition to the 15 already synthesized (Table 3, named HPV30-38) and evaluated the efficiency predictions as in the previous step (3E30 model, Table 4). As the most efficient DNAzymes had already been tested in the previous experiments, this pool of 8 DNAzymes was biased towards those that were less efficient. Cleavage reactions demonstrated that these DNAzymes had varying degrees of efficiency (Supplementary Figure S1b). Accuracy was similar to the previous model (62.5%) and precision increased (50%), although recall decreased to 33% due to one specific DNAzyme with relatively high efficiency (HPV36) that was not predicted by the logistic regression (see Discussion).

**Table 5.** Logistic regression of thermodynamic properties and efficiencies for 30 DNAzymes

	df	Deviance	Residual df	Residual deviance	*P*-value
Null			29	34.8	
Binding energy	1	6.9	28	27.8	0.008
Dimer energy	1	0.05	27	27.8	0.825
Internal energy	1	6.2	26	21.6	0.012

By incorporating all 38 DNAzymes assessed in this work, the logistic regression model provides strong evidence that binding and internal energies influence the DNAzyme efficiency (Table 6). Importantly, as we add additional data on DNAzymes to the model, it becomes more robust, and the predictions improve. Putting all our findings together, we proposed an iterative method for the rational identification of DNAzymes.

**Table 6.** Logistic regression of thermodynamic properties and efficiencies for all 38 DNAzymes

	df	Deviance	Residual df	Residual deviance	*P*-value
Null			37	45.7	
Binding energy	1	6.3	36	39.4	0.012
Dimer energy	1	0.8	35	38.5	0.358
Internal energy	1	4.2	34	34.3	0.040

### A method to find potentially efficient DNAzymes in custom targets

**Figure 4.**
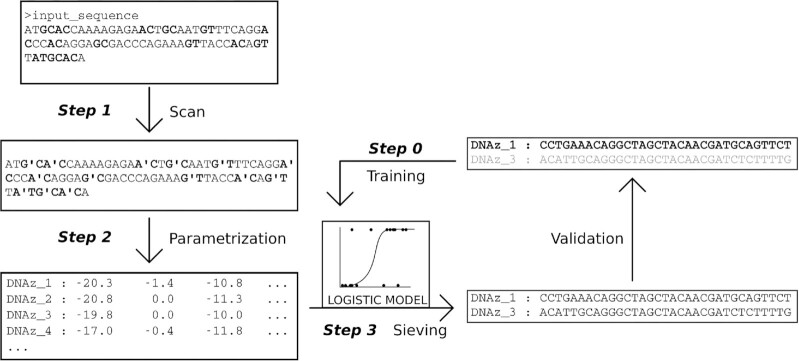
Overview of the proposed computational method. This cartoon provides a graphic summary of the process used to identify DNAzymes as described in the main text (see Results for details).

Here, we propose a method to build efficient 10–23 DNAzymes based on our findings about the relationship between binding energy and efficiency. The algorithm (illustrated in Figure 4) runs as follows:

***Step (0* *) Training (optional)***. Efficiency values for various DNAzymes can be provided (as we do above) to build a logistic model specific to the target sequence. This step is optional, but it can be useful to improve the model as more data on DNAzyme efficiencies become available. Users can therefore progressively refine their models.
***Step (1* *) Scan*.** The algorithm scans the input RNA sequence looking for Purine:Pyrimidine (RY) junctions, which are potential cleavage sites. A potential 10–23 DNAzyme targeting each site is reconstructed with a custom arm length (9 nucleotides by default) and the catalytic core.
***Step (2* *) Parametrization*.** For each potential DNAzyme, a number of parameters are computed. In the current version proposed, the algorithm computes a predicted binding energy based on a nearest-neighbour algorithm, as well as homodimer and internal structure folding energies (see Materials and Methods for software and details), but other parameters can be easily added.
***Step (3* *) Sieving*.** The parameter(s) from the previous step are fed into the pre-built logistic model (provided or generated in step 0). The algorithm will provide a list of DNAzymes that potentially cleave the RNA input sequence at the pre-set efficiency level.

By following these simple steps and the software we provide, researchers can iteratively refine their predictions or benefit from already published data to generate efficient DNAzymes in a rational way, without extensive selection screens of random candidate molecules.

## DISCUSSION

The development of 10–23 DNAzymes has been hampered by the lack of tools that can predict DNAzyme efficiency and/or assist with DNAzyme identification. Here, we propose a method for the rational construction of RNA transcript-cleaving 10–23 DNAzymes that complements the work in the laboratory to identify the most efficient DNAzymes specific to an RNA target, generally transcripts. To develop this model, we used HPV16 E6/E7 RNA as a target, and first evaluated the DNAzymes previously proposed and tested by Cairns *et al.* (28). Cairns *et al.* previously assessed 80 DNAzymes targeting this transcript (28). For our training dataset, we assessed the efficiency of 15 of these DNAzymes. In agreement with Cairns *et al.*, we found DT99 to be the most efficient DNAzyme under single turnover conditions (10-fold excess of DNAzyme to RNA) using the long transcript, cleaving almost 80% of the target within 60 min. The efficiencies generated were subsequently used to explore the importance of three different Δ*G* energies in predicting DNAzyme efficiency: hybridization free energy (Δ*G*), dimer formation energy (intermolecular structures) and hairpin formation (intramolecular structures) energy.

The significance of Δ*G* or hybridization free energy was previously highlighted by Cairns *et al.* (28). They showed that it is possible to assess DNAzyme efficiency by plotting the predicted Δ*G* values for DNAzymes against RNase activity profiles in order to determine the relationship between heteroduplex stability and DNAzyme efficiency (28). Cairns *et al.* suggested a value of lower than −20 kcal/mol being important in determining DNAzyme efficiency in long transcripts; however, they also stated that Δ*G* alone was not enough to predict DNAzyme efficiency (28). In agreement with this, we also found that DNAzymes with an energy lower than −20 kcal/mol were generally usually efficient. However, there were exceptions to this rule, suggesting that other parameters also affect DNAzyme efficiency.

A large negative value of free energy for the DNAzyme internal structure indicates a stable and therefore undesirable hairpin structure with Cairns suggesting a value of no less than −2 kcal/mol being beneficial to DNAzyme efficiency (28,29). We also found that low hairpin energy correlates with inefficient DNAzymes, with our efficient DNAzymes (>40% cleavage) having an internal energy of > −1 kcal/mol. Internal energy should therefore be taken into account when considering novel DNAzymes. The formation of DNAzyme dimers will reduce the concentration of available DNAzymes in a cleavage reaction, similar to hairpin formation. Further, dimers can also result in extended DNAzyme products that are unwanted in a reaction (28). However, in contrast to internal energy, we did not find a correlation between dimer energy and DNAzyme efficiency. We are still investigating this in our laboratory.

Using the DNAzyme efficiency data, a multiple logistic regression model was developed, taking into account Δ*G*, hairpin and dimer energies. Importantly, this was able to successfully identify HPV16 E6/E7-targeting DNAzymes with high efficiency. For instance, HPV24 has an efficiency of 62.2%. However, we also identified via *in vitro* testing a DNAzyme (HPV36) with high efficiency that our current model did not predict to be efficient. In this case, the dimer energy for this DNAzyme was low, and it is therefore possible that the model interprets this as an issue against high efficiency.

To reconcile these issues, future implementation of the method can include other parameters accounting for nucleotide content biases or specific motif signatures. This is possible since the logistic regression can be modeled to include not only continuous and ranked variables but also categorical variables. For instance, in the computational scan of siRNA molecules there are additional factors that can be taken into account, such as internal structure of the target RNA (36) and specific nucleotide composition (37), among others [reviewed in (38)]. A specific feature of DNAzymes that can also be considered is the preference of certain purine:pyrimidine junctions over other combinations (18,28). We specifically focused here on those junctions reported to be the most efficient (GU and AU), but future models may incorporate other combinations to study the joint effect of purine:pyrimidine junctions and other features.

Several other parameters/factors are also likely to affect DNAzyme efficiency, and these should also be taken into consideration when selecting DNAzymes for testing. For example, the folding of the target RNA is likely to play an important role in DNAzyme efficiency. However, the effect of RNA folding has not been systematically explored in relation to DNAzymes and further work is therefore needed to assess this. Another factor that will affect cleavage efficiency is substrate binding arm length. The longer the arm length, the more specific and stable that the interaction will be. However, longer arm lengths are more likely to form secondary structures, and/or increase heteroduplex formation, potentially reducing cleavage efficiency. Santoro and Joyce first described an increased efficiency for DNAzymes with substrate binding arms over 4 nt (39). Importantly, it was noted that this increase in efficiency increased significantly in DNAzymes with an arm length of 4–7 nt, but this slowed when binding arm length was increased to 7–13 nt (39,40). In agreement with this, our data indicate that DNAzymes with 9 nt substrate binding arms perform similarly to DNAzymes with longer arm lengths.

A balance between specificity, the strength of the heteroduplex formation and secondary structures therefore needs to be found. As the number of parameters and permutations increase exponentially, heuristic algorithms will need to be implemented to consider these variables. As additional data regarding DNAzyme efficiency are generated experimentally, this information can be fed into the analysis of these parameters, increasing the accuracy of our predictions.

This work is a first step in the development of tools to identify efficient RNA-cleaving DNAzymes. Also, it is an important step towards understanding parameters involved in heteroduplex formation and stability, and whether these affect 10–23 DNAzyme catalytic activity. This can only be determined by evaluating myriads of DNAzymes under different assay conditions, and in terms of using DNAzymes as potential therapeutics, in different cellular contexts. However, we do recognize that the number of DNAzymes used in the study is relatively low and the future inclusion of additional cleavage efficiencies will strengthen the predictions. Further, it should be noted that the fluorescent labelling of the target RNA may have had an effect upon cleavage efficiency. Future analysis of non-labelled target RNA will resolve this potential issue. Here, we focused on 10–23 DNAzymes due to availability of published cleavage efficiency data and also due to the therapeutic potential of these molecules. Importantly, this method could be modified to evaluate other types of molecules, such as 8–17 DNAzymes (41,42) and/or chemically modified versions (43). A standard tool for identifying and evaluating the properties of RNA-cleaving DNAzymes, such as the one proposed here, is an important step in the development of this technology and will help to accelerate the potential impact of these molecules.

## DATA AVAILABILITY

Scripts to reproduce the statistical analyses in the paper, as well as a script to scan RNA sequences for potential DNAzymes, are publicly available from GitHub at https://github.com/antoniomarco/DNAzymes_10_23 and Zenodo at https://doi.org/10.5281/zenodo.7415194.

## Supplementary Material

lqac098_Supplemental_File
