# Supplementary material for: A computational approach to identify efficient RNA cleaving 10–23 DNAzymes
Source: NAR Genom Bioinform. 2023 Jan 10;5(1):lqac098. doi: 10.1093/nargab/lqac098 (PMC9830538; doi:10.1093/nargab/lqac098)
Supplement: lqac098_Supplemental_File [file lqac098_supplemental_file.pdf]

**Supplemental Figure 1**

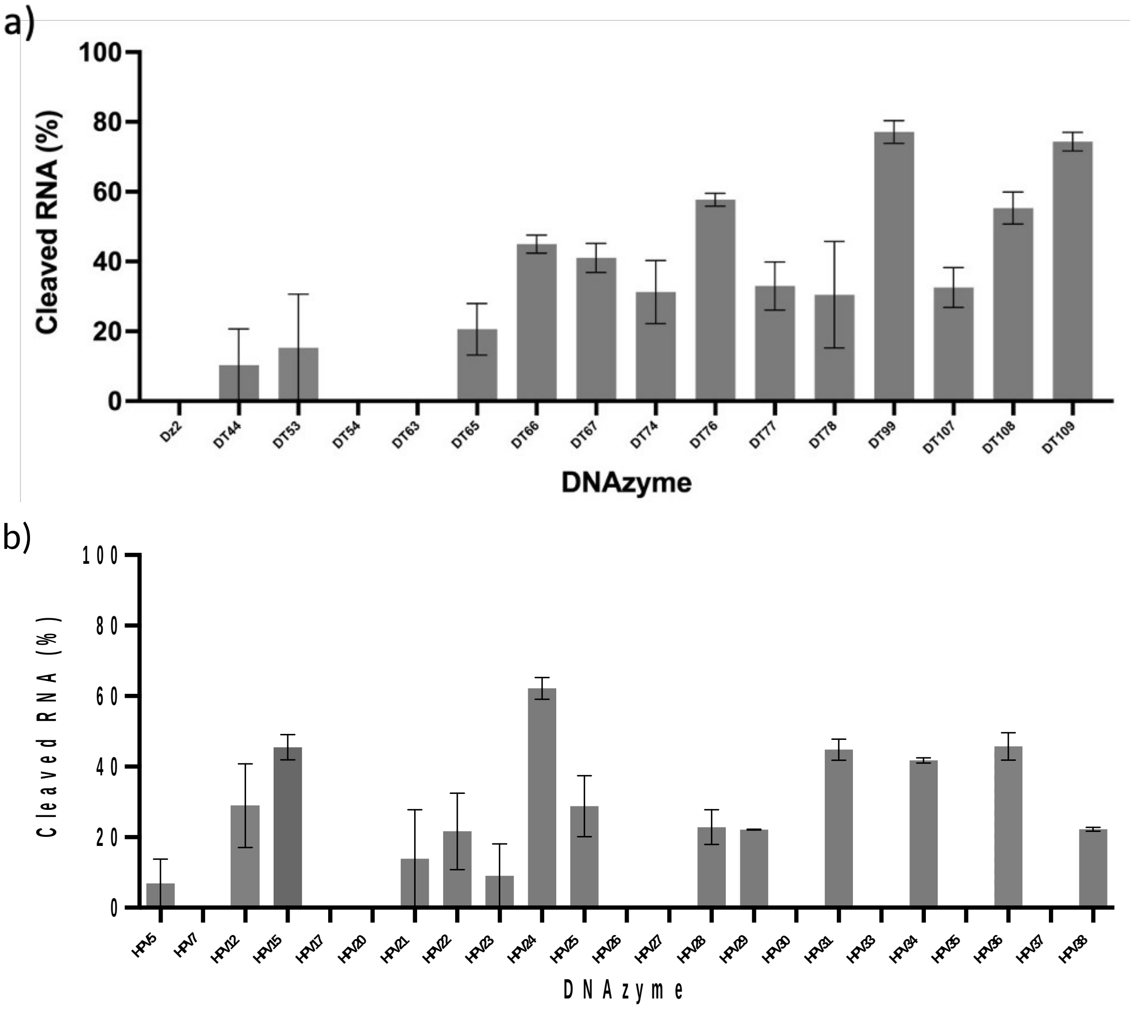


**Supplemental Figure 1. Cleavage efficiency of HPV16 E6/E7 targeting DNAzymes.** HPV16 E6/E7 DNAzymes, a) previously published by Cairns et al. and b) designed using the computational tool, were incubated separately from FITC labelled *in vitro* transcribed HPV16 E6/E7 RNA at a ratio of 10:1 in the DNAzyme buffer at 37°C prior to combination and further incubation for 60 minutes. Reactions were stopped through the addition of loading dye and snap freezing. Samples were separated by urea PAGE and densitometry performed using Image J. Mean of 3 independent repeats ± SE

**Supplemental Figure 2**

a)

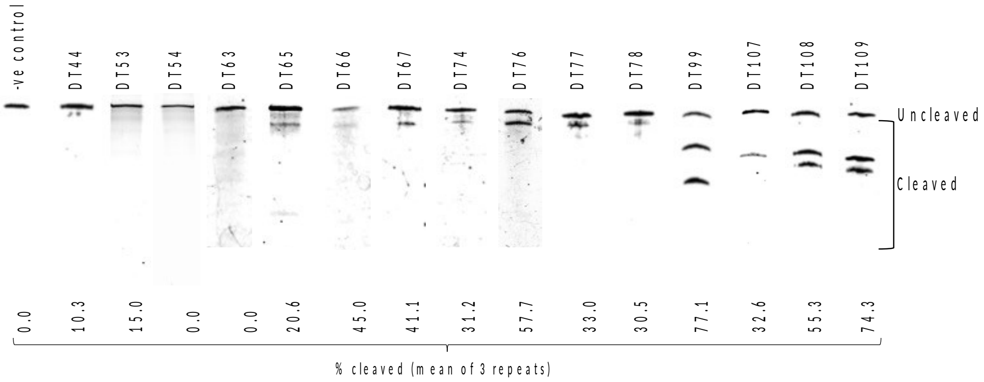


b)

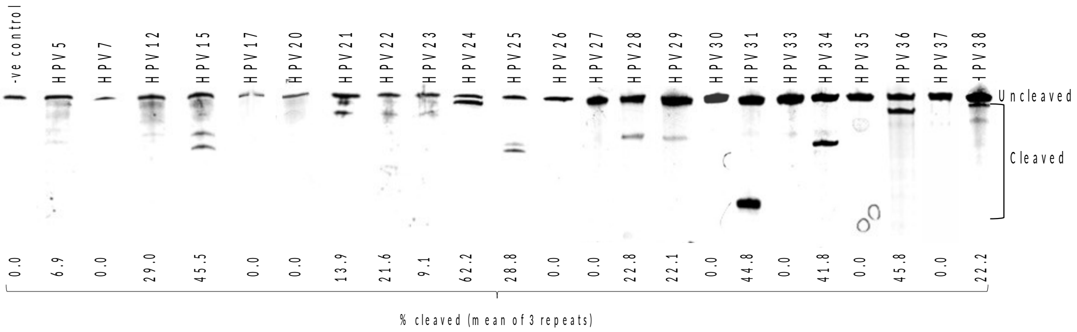


**Supplemental Figure 2. Representative examples of urea PAGE gels for HPV16 E6/E7 targeting DNAzymes.** HPV16 E6/E7 DNAzymes, a) previously published by Cairns et al. and b) designed using the computational tool, were incubated separately from FITC labelled *in vitro* transcribed HPV16 E6/E7 RNA at a ratio of 10:1 in the DNAzyme buffer at 37°C prior to combination and further incubation for 60 minutes. Reactions were stopped through the addition of loading dye and snap freezing. Samples were separated by urea PAGE and densitometry performed using Image J. A representative image has been provided. The percentages are the mean of 3 independent repeats.

**Supplemental Figure 3**

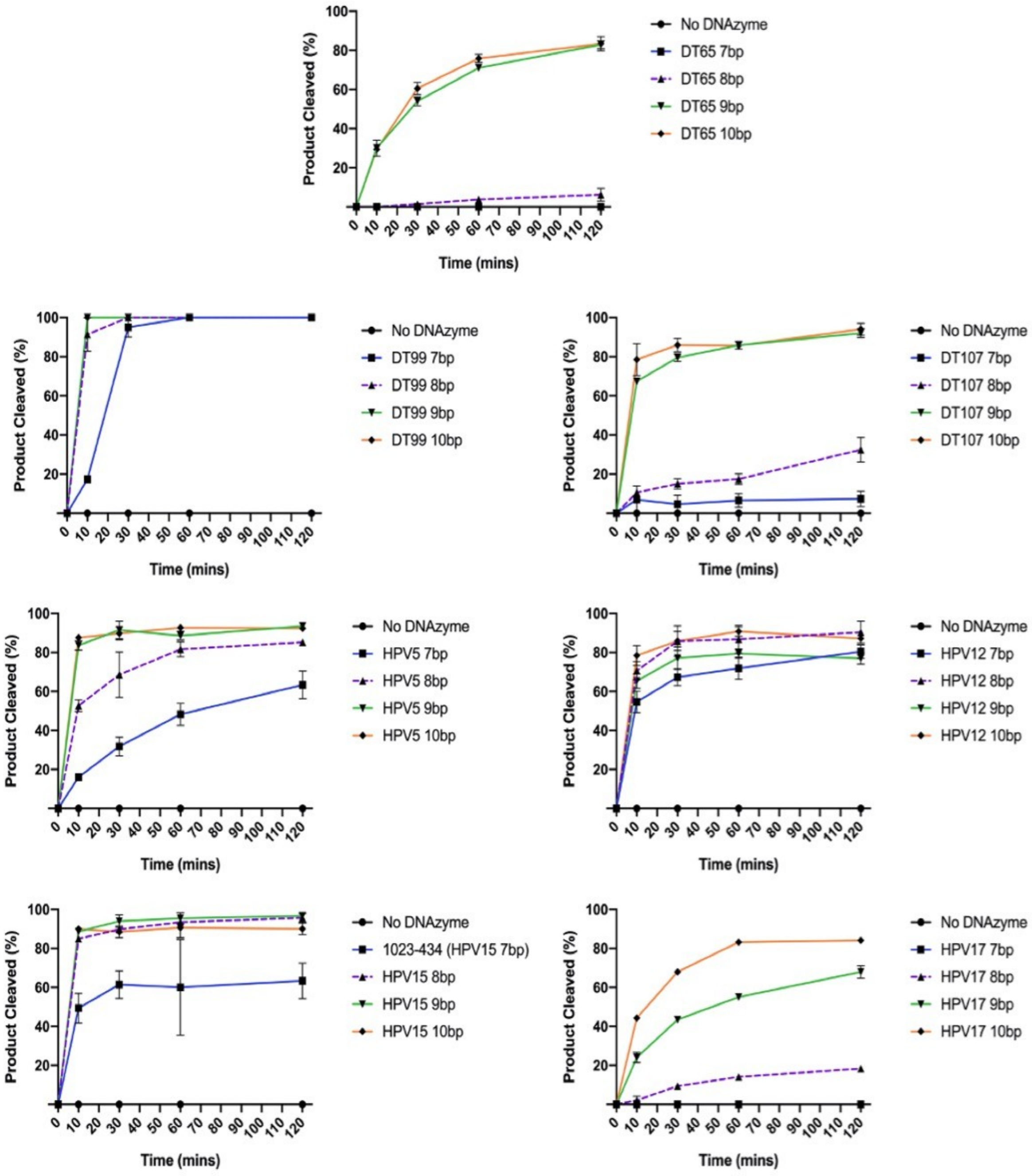


**Supplemental Figure 3. DNAzymes with 9 nt binding arms are efficient.** 0.1 mM FAM labelled target RNA and 0.25 mM DNAzymes with substrate binding arms between 7-10 nt were incubated separately in DNAzyme buffer containing 10 mM of $Mg^{2+}$ for 10 min at 37 ºC. DNAzymes were then combined and incubated with target FAM RNA fragments at 37 ºC for up to 120 min. Reactions were stopped by the addition of loading dye at time intervals of 0, 10, 30, 60 and 120 mins. The samples were separated by urea PAGE. ImageJ was used to perform densitometry on the resulting product bands. Mean of 3 independent repeats ±SE.

**Supplemental Table 1. The effect of substrate binding arm length upon cleavage efficiency.** 0.1 mM FAM labelled target RNA and 0.25 mM DNAzymes with substrate binding arms between 7-10 nt at 37 ºC for up to 120 min. Samples were separated by urea PAGE and densitometry was performed using ImageJ. Mean of 3 independent repeats ±SE.

| Dz* | Binding Arm Length | Sequence** | Efficiency (%) at 10 min ±SEM | Efficiency (%) at 30 min ±SEM | Efficiency (%) at 60 min ±SEM | Efficiency (%) at 120 min ±SEM |
|---|---|---|---|---|---|---|
| HPV5 | 7 | CAGGACA[CC]AGTGGCT | 16.1±1.7 | 31.8±4.9 | 48.3±5.7 | 63.3±7.2 |
| | 8 | TCAGGACA[CC]AGTGGCTT | 52.7±3.0 | 68.5±11.7 | 81.7±3.8 | 85.2±1.8 |
| | 9 | TTCAGGACA[CC]AGTGGCTTT | 83.6±2.4 | 91.5±4.5 | 88.6±2.9 | 93.6±1.4 |
| | 10 | CTTCAGGACA[CC]AGTGGCTTTT | 87.6±1.8 | 89.8±3.3 | 92.7±1.5 | 92.4±0.6 |
| HPV12 | 7 | AGACATA[CC]ATCGACC | 54.6±5.6 | 67.3±4.4 | 71.9±5.7 | 80.5±3.4 |
| | 8 | AAGACATA[CC]ATCGACCG | 70.6±4.7 | 85.8±7.9 | 86.8±7.1 | 90.4±5.7 |
| | 9 | CAAGACATA[CC]ATCGACCGG | 65.4±3.7 | 77.2±5.8 | 79.5±2.3 | 76.9±3.0 |
| | 10 | ACAAGACATA[CC]ATCGACCGGT | 78.5±5.1 | 86.0±4.9 | 90.8±2.4 | 87.2±1.0 |
| HPV15 | 7 | TTCAGGA[CC]ACAGTGG | 37.9±0 | 71.3±0 | 78±0 | 81.5±0 |
| | 8 | CTTCAGGA[CC]ACAGTGGC | 84.9±1.1 | 89.8±0.3 | 93.5±0.3 | 95.7±2.3 |
| | 9 | TCTTCAGGA[CC]ACAGTGGCT | 88.6±1.8 | 94.0±3.3 | 95.5±2.9 | 96.7±1.8 |
| | 10 | TTCTTCAGGA[CC]ACAGTGGCTT | 90.0±1.1 | 88.5±3.0 | 90.6±5.1 | 90.0±3.0 |
| HPV17 | 7 | CAGCATA[CC]GGATTCC | ±0 | 0±0 | 0±0 | 0±0 |
| | 8 | ACAGCATA[CC]GGATTCCC | 2.1±0 | 9.4±1.4 | 14.1±1.0 | 18.4±1.6 |
| | 9 | TACAGCATA[CC]GGATTCCCA | 24.0±2.6 | 43.5±0.9 | 55.1±2.0 | 67.9±3.2 |
| | 10 | ATACAGCATA[CC]GGATTCCCAT | 44.3±1.3 | 68.0±1.2 | 83.2±1.3 | 84.1±2.0 |
| DT65 | 7 | ATATGGA[CC]TCCCATC | 0±0.0 | 0±0.0 | 0±0.0 | 0±0.0 |
| | 8 | CATATGGA[CC]TCCCATCT | 0±0.0 | 1.4±1.4 | 3.8±1.9 | 6.2±3.3 |
| | 9 | GCATATGGA[CC]TCCCATCTC | 30.0±4.1 | 54.0±2.3 | 71.0±2.0 | 82.9±2.0 |
| | 10 | AGCATATGGA[CC]TCCCATCTCT | 29.4±1.3 | 60.6±3.1 | 75.8±2.2 | 83.3±3.6 |
| DT99 | 7 | TTCTCTA[CC]GTGTTCT | 25.5±8.1 | 91.1±4.9 | 97.8±2.2 | 100±0.0 |
| | 8 | TTTCTCTA[CC]GTGTTCTT | 94.3±5.7 | 100±0.0 | 100±0.0 | 100±0.0 |
| | 9 | GTTTCTCTA[CC]GTGTTCTTG | 100±0.0 | 100±0.0 | 100±0.0 | 100±0.0 |
| | 10 | GGTTTCTCTA[CC]GTGTTCTTGT | 100±0.0 | 100±0.0 | 100±0.0 | 100±0.0 |
| DT107 | 7 | ACATCGA[CC]CGGTCCA | 7.0±7.0 | 4.6±4.6 | 6.5±3.5 | 7.4±3.4 |
| | 8 | TACATCGA[CC]CGGTCCAC | 10.7±1.0 | 15.0±2.6 | 17.5±2.6 | 32.5±6.3 |
| | 9 | ATACATCGA[CC]CGGTCCACC | 67.4±1.1 | 79.5±2.0 | 85.4±2.0 | 92.1±2.2 |
| | 10 | CATACATCGA[CC]CGGTCCACCG | 78.6±8.2 | 86.0±3.4 | 85.7±1.6 | 94.0±3.0 |

* DNAzyme; **[CC] : 10-23 DNAzyme catalytic core (GGCTAGCTACAACGA)

**Supplemental Table 2.**
Raw values used to compute accuracy, precision and recall of the models.

| | 1E15 | 3E15 | 3E30 |
|---|---|---|---|
| True Positives (TP) | 1 | 1 | 1 |
| False Positives (FP) | 3 | 4 | 1 |
| True Negatives (TN) | 10 | 9 | 4 |
| False Negatives (FN) | 1 | 1 | 2 |
| TOTAL | 15 | 15 | 8 |
